# Impact of Pasteurization and Storage on the Microbiological Composition and Lipid Degradation of Human Milk Cream

**DOI:** 10.3390/foods15112025

**Published:** 2026-06-04

**Authors:** Diana Escuder-Vieco, Juan M. Rodríguez, Leónides Fernández, María Visitación Calvo, Javier Fontecha, Diana Martín, Kristin Keller, José Luis Carrión-Frías, Carmen R. Pallás-Alonso, Nadia R. García-Lara

**Affiliations:** 1 Aladina-MGU-Regional Human Milk Bank, 12 de Octubre University Hospital, imas12, 28041 Madrid, Spain; biol.kristin.keller@gmail.com (K.K.); kpallas.hdoc@gmail.com (C.R.P.-A.); nadiaraquelg.nrgl@gmail.com (N.R.G.-L.); 2 Sección Departamental de Nutrición y Ciencia de los Alimentos, Universidad Complutense de Madrid, 28040 Madrid, Spain; jmrodrig@vet.ucm.es; 3 Instituto Pluridisciplinar, Universidad Complutense de Madrid, 28040 Madrid, Spain; leonides@vet.ucm.es; 4 Sección Departamental de Farmacia Galénica y Tecnología Alimentaria, Universidad Complutense de Madrid, 28040 Madrid, Spain; 5 Food Lipid Biomarkers and Health Group, Institute of Food Science Research (CIAL), CSIC-UAM, 28049 Madrid, Spain; mv.calvo@csic.es (M.V.C.); j.fontecha@csic.es (J.F.); 6 Departamento de Producción y Caracterización de Nuevos Alimentos, Instituto de Investigación en Ciencias de la Alimentación (CIAL) (CSIC–UAM), 28049 Madrid, Spain; diana.martin@uam.es; 7 Sección Departamental de Ciencias de la Alimentación, Facultad de Ciencias, Universidad Autónoma de Madrid, 28049 Madrid, Spain; 8 Department of Microbiology, 12 de Octubre University Hospital, imas12, 28041 Madrid, Spain; jcarrion@salud.madrid.org; 9 Department of Neonatology, 12 de Octubre University Hospital, imas12, 28041 Madrid, Spain

**Keywords:** breast milk, cream, pasteurization, microbiology, lipolysis, oxidation, fortification, preterm infants

## Abstract

Human milk cream, a lipid-rich fraction obtained during milk defatting, is typically discarded despite its potential for individualized nutritional strategies. This study evaluated the effects of Holder pasteurization (HoP) and storage conditions (refrigeration and freezing) on the microbiological profile and lipid stability of human milk–derived cream. Cream fractions from six mothers of preterm infants were analyzed for bacterial counts using Columbia Nalidixic Acid and MacConkey agar, with isolate identification by MALDI-TOF MS, and for lipid stability through free fatty acids (FFAs), triacylglycerol (TAGs) composition by GC-FID, and peroxide values (PV) determined by a rapid photometric method. Raw cream showed stable total bacterial counts across storage conditions, with a reduction in Gram-negative bacteria after freezing, while HoP samples exhibited no detectable bacterial growth. Lipolysis was significantly higher in raw cream, with increased FFAs after 72 h refrigeration, whereas HoP samples maintained lower and more stable FFA levels. TAG profiles remained largely stable under refrigeration but were significantly altered during frozen storage in raw samples, suggesting membrane disruption and selective hydrolysis; these changes were attenuated in HoP samples. PV increased over time in both groups, indicating progressive primary lipid oxidation, although values remained within moderate ranges. Overall, HoP improved microbiological safety and lipid stability, supporting the potential use of human milk cream as a controlled lipid source for individualized fortification in preterm infants.

## 1. Introduction

Mother’s own milk (MOM) constitutes the gold standard for preterm infant nutrition due to its complex composition of macronutrients, bioactive compounds, and immunological factors, while donor human milk (DHM) represents the best option when MOM is unavailable [1].

In specific clinical settings, tailored modifications of the human milk composition may be required to accommodate metabolic constraints and individualized nutritional strategies. In this context, skimmed human milk (SHM), either from MOM or DHM, has emerged as a viable nutritional strategy for infants requiring dietary fat restriction, including those with neonatal conditions such as congenital chylothorax [2], congenital nephrotic syndrome [3], or inborn errors of long-chain fatty acid oxidation [4,5]. In such cases, SHM enables the continuation of human milk feeding since it retains many of the biologically relevant properties of this biological fluid [6,7].

Human milk defatting by refrigerated centrifugation has been identified as a highly efficient and reproducible method [8], yielding two distinct fractions: a lipid-depleted aqueous phase (SHM) and a lipid-rich cream fraction containing the majority of triacylglicerols (TAGs), phospholipids, and fat-soluble micronutrients. The resulting cream fraction, often referred to as the “cream layer,” is frequently discarded in clinical practice; however, it represents a concentrated, energy-dense component with potential applications in individualized nutritional strategies for neonates requiring a tailored provision of lipids.

Lipid enrichment of human milk may enhance short-term weight gain in preterm populations, although the certainty of evidence varies depending on the intervention and study design [9]. Recently, the incorporation of human milk fat modular to feeding protocols for very low birth weight infants have been associated with better growth outcomes, reduced rates of malnutrition, and decreased reliance on higher-calorie fortifiers, while maintaining an exclusive human milk-based diet (EHMD) [10]. The development of human milk-derived lipid ingredients is particularly relevant because it may reduce the exposure of preterm infants to bovine-derived or other non-human milk supplements, while better preserving the biological and nutritional specificity of human milk. Despite these potential clinical benefits, the implementation of lipid-based nutritional strategies remains constrained by cost, limited availability, and the need for well-controlled processing and storage of human milk–derived components.

The human milk microbiota is present in whole and skimmed milk but, also, in the fat layer collected after defatting the milk [11]. In fact, it has been observed that bacteria cannot be removed from the cream fraction of human milk by centrifugation [12]. However, studies on the microbial composition of human milk fat and the influence of processing such milk fraction are almost absent. In addition, the cream fraction of this biological fluid is characterized by a modified phase distribution, and a specific interfacial organization governed by the milk fat globule membrane (MFGM), a complex trilayer structure rich in polar lipids and proteins that plays a key role in emulsion stability and enzyme interactions [13]. The integrity of this membrane critically regulates the accessibility of endogenous lipases and the susceptibility of milk lipids to hydrolytic and oxidative degradation [14]. Recent evidence suggests that the MFGM is highly sensitive to both thermal processing and freeze–thaw cycles. Freezing may induce ice crystal formation, fat globule aggregation, and phase separation, leading to partial disruption of the interfacial membrane and increased exposure of core lipids to lipases and pro-oxidant compounds [15,16]. Similarly, thermal treatments may alter the protein and polar lipid composition of the MFGM, thereby affecting fat globule stability and lipid reactivity during storage [14]. In human milk, frozen storage has been associated with physicochemical and lipidomic alterations, including changes in lipid organization and reduced stability of specific lipid classes [15,16]. These observations support the hypothesis that freeze–thaw conditions may modify fat globule organization and increase lipid accessibility to endogenous enzymes.

In addition, Davis and Perrin in a scoping review [17] indicated that inconsistencies in reported fat losses following pasteurization may stem from methodological differences, particularly pre-analytical sample mixing, underscoring the need for standardized handling protocols when evaluating cream lipid composition.

Evidence from bovine dairy systems further supports the high susceptibility of fat-rich dairy matrices to storage-related lipid degradation. Refrigeration has been associated with increased lipoprotein lipase (LPL) activity in cream, promoting TAGs hydrolysis and the accumulation of free fatty acids (FFAs), which are highly prone to oxidative degradation [18]. Peroxide value (PV) is widely used as an indicator of primary lipid oxidation in dairy fat systems, including cream and butter, where relatively low PVs are generally associated with early-stage oxidation under controlled storage conditions [19,20].

However, studies specifically addressing lipid degradation and oxidative stability in human milk cream during processing and storage remain very scarce. Therefore, this work aimed to assess the effects of Holder pasteurization (HoP) and different storage conditions (refrigeration and freezing) on the microbiological profile and lipid degradation of human milk-derived cream.

## 2. Materials and Methods

### 2.1. Human Milk Samples

Human milk samples were provided by six mothers of preterm infants admitted to the Neonatology Unit at Hospital Universitario 12 de Octubre (Madrid, Spain). Gestational age at delivery ranged from 26 to 36 + 5 weeks (mean: 33 weeks), and milk contributing to the pooled samples was collected at a mean postnatal age of 38 days postpartum (range: 8–73 days). Milk was obtained using electric breast pumps (Medela Symphony, Medela AG, Baar, Switzerland) into sterile containers and immediately frozen (−25 °C) in hospital freezers. Samples were then transported to the human milk bank in the same hospital using insulated coolers equipped with cold packs. The study was conducted in accordance with the principles outlined in the Declaration of Helsinki (1975, revised in 2013), and approved by the Clinical Research Ethics Committee of “Hospital 12 de Octubre” (protocol code 21/202). All participants signed a written informed consent declaration.

### 2.2. Experimental Design

**Figure 1:**
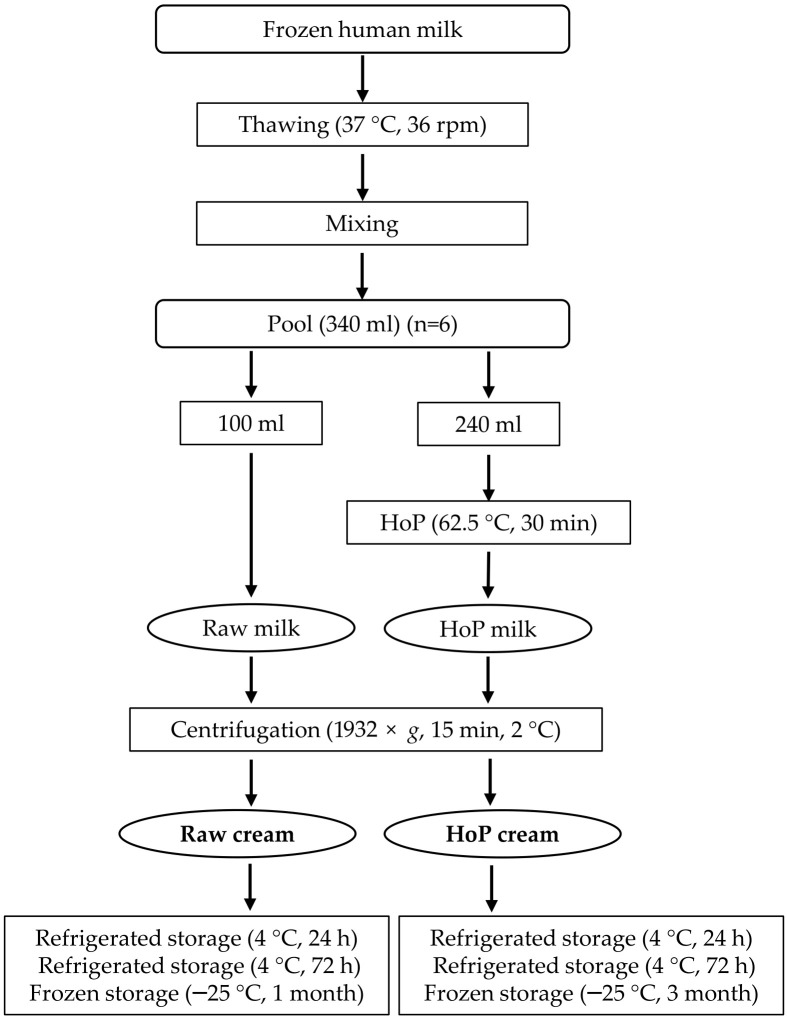
Experimental design and storage conditions of human milk cream samples. HoP—Holder pasteurization.

After collection, human milk samples (*n* = 6) were kept frozen (−25 °C) for a period ranging between 14 and 47 days (mean: 26 days). Then, the samples were thawed in a shaking water bath (36 rpm) at 37 °C. Milk from each mother was pooled in a sterile flask within a laminar-flow cabinet (Fortuna 1200 Maxi; Controltecnica SL, Madrid, Spain) to obtain a final volume of approximately 340 mL. One aliquot (100 mL) was kept raw (unheated), while the remaining portion (~240 mL) was subjected to standard Holder pasteurization (HoP; 62.5 °C, 30 min) in a shaking (36 rpm) water bath. Both raw and HoP milk samples were centrifuged at 1932× *g* for 15 min at 2 °C (Eppendorf Centrifuge 5910Ri, Eppendorf, Iberica S.L.U, Madrid, Spain). After centrifugation, the cream layer was carefully isolated by making a small opening in the fat layer and removing the underlying skimmed milk using a sterile syringe (Pentaferte Italia Srl, Campli, Italy) fitted with a feeding tube (ref. 533.10, 10 Fr, 3.3 mm diameter; Vygon, Ecouen, France). The cream fraction was then aseptically transferred using sterile spatulas into sterile 8 mL clear glass vials (Wheaton, Millville, NJ, USA). Aliquots of 0.5 g and 0.8 g were prepared for raw and HoP cream samples, respectively. Raw cream samples were stored under refrigeration (4 °C) for 24 h and 72 h and, in parallel, frozen (−25 °C) for one month, while HoP cream samples were stored under the same refrigerated conditions and frozen for up to three months (Figure 1). The selected storage periods were designed to reflect routine clinical practice in human milk banks, where raw donor milk is generally stored frozen for a limited period before pasteurization (commonly 4–6 weeks), whereas HoP milk may be stored frozen for up to 3 months before use. Accordingly, the longer frozen-storage period applied to HoP cream was intended to explore the potential of pasteurization to extend storage stability.

### 2.3. Microbiological Analysis

Raw and HoP cream samples were diluted using sterile peptone water and spread onto agar plates of a general medium for Gram-positive bacteria (Columbia Nalidixic Acid agar; CNA; bioMérieux) and a selective medium for the enumeration and isolation of Enterobacterales and other Gram-negative bacteria (MacConkey agar; MCK; bioMérieux).

After an incubation at 37 °C for 48 h under aerobic conditions, the colonies were enumerated and the most abundant bacterial colony types, based on morphological characteristics (color, size, shape, and presence of halos), observed on each medium were selected for identification. Identification was carried by matrix-assisted laser desorption/ionization time-of-flight mass spectrometry (MALDI-TOF MS) using the VITEK^®^ MS system (bioMérieux) at the Microbiology Service of Hospital 12 de Octubre (Madrid, Spain).

### 2.4. Determination of Free Fatty Acids (FFAs) and Triacylglycerols (TAGs) by GC-FID

Lipid extraction was performed following the simplified method of [21]. FFAs and TAGs molecular species by carbon number were identified and quantified by gas chromatography with a Clarus 400 GC (PerkinElmer Ltd., Beaconsfield, UK) equipped with a split/splitless injector and FID, following the experimental conditions used by [22]. After dissolving the lipid extracts in dichloromethane (20 mg/mL), they (0.5 μL) were injected into a capillary column (Rtx-65TAG; 30 m × 0.25 mm i.d. × 0.1-μm film thickness; Restek Corp., Bellefonte, PA, USA) and the quantification of TAGs molecular species was performed on the basis of their number of carbon atoms (CN). An anhydrous milk fat with a known TAGs composition (reference material BCR-519; EU Commission, Brussels, Belgium; purchased from Fedelco Inc., Madrid, Spain) was employed to calculate the response factors required for the qualitative and quantitative analysis.

### 2.5. Determination of Peroxide Values

Peroxide value (PV) determination was carried out immediately after lipid extraction. A FoodLab photometric analyzer (CDR S.r.L., Ginestra Fiorentina, Italy) for PV, considered equivalent to the AOCS Official Method Cd 8–53, was used, following the procedure described by Martín et al. (2012) [23]. The sample (5 µL) was reacted with the commercial reagents supplied by the instrument manufacturer. After incubation at 37 °C for 3 min in the thermostatically controlled chamber of the instrument, absorbance was recorded at 505 nm. Quantification was performed using the calibration parameters integrated into the FoodLab system. All samples were analyzed in duplicate.

### 2.6. Statistical Analysis

Microbial counts were expressed as log_10_ (colony-forming units [CFU]/mL) values. FFAs and TAGs concentrations were expressed as g/100 g fat. PVs were recorded as milliequivalents of active oxygen per kilogram of fat (mEq O_2_/kg). Results are displayed as mean (standard deviation—SD).

Non-parametric tests were applied because of the small sample size (*n* = 6). Differences in microbial counts, FFAs, TAGs, and PV across storage conditions were evaluated using the Friedman test for repeated measures, with effect size estimated by Kendall’s coefficient of concordance (W). When significant effects were detected, *post hoc* pairwise comparisons between storage conditions or between raw and HoP samples at each time point were assessed employing the Wilcoxon signed-rank test for paired samples. Statistical analyses were conducted with SPSS 26 software (IBM SPSS Statistics Inc., Chicago, IL, USA), and the significance threshold was set at *p* < 0.05.

## 3. Results

### 3.1. Microbiological Composition

Microbial counts in raw and HoP cream samples are presented in Table 1. Overall, total counts [mean (SD)] in raw cream samples were 3.85 (1.05) log_10_ CFU/mL after 24 h of refrigeration, 3.42 (0.97) log_10_ CFU/mL after 72 h of refrigeration, and 3.31 (1.15) log_10_ CFU/mL after one month of freezing. No significant differences in total bacterial counts (Friedman test, χ^2^ = 5.48, *p* = 0.065) and bacterial counts on CNA (χ^2^ = 1.83, *p* = 0.401) across storage conditions.

However, a significant effect was observed for bacterial counts on MCK medium (χ^2^ = 7.00, *p* = 0.030), with post hoc analysis indicating a significant difference between bacterial counts after 24 h of refrigeration and frozen storage (*p* = 0.031). No bacterial growth was detected in HoP cream samples after refrigeration or frozen storage (Table 1).

In relation to the bacteria detected on CNA plates, *Staphylococcus epidermidis* was the most frequent species since it was isolated from four samples (66.7%), while *Enterococcus faecalis* and *Pseudomonas fulva* were only detected in one sample (16.7%). In the case of MCK agar, *Serratia liquefaciens* was present in three samples (50.0%), while other bacterial species (*P. fulva, Escherichia coli*, and *Acinetobacter ursingii*) were identified only in one sample (16.7%).

### 3.2. Analysis of FFAs and TAGs by GC-FID

**Figure 2:**
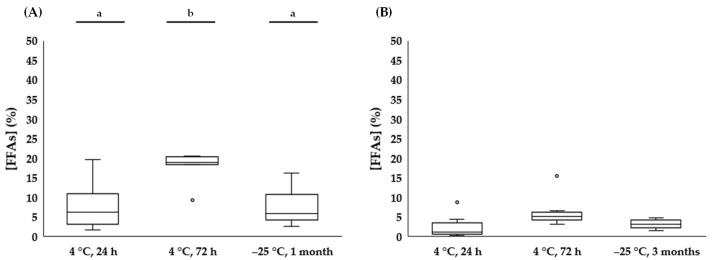
Free fatty acids (FFAs) concentration (g/100 g fat) in raw (**A**) and Holder pasteurization (**B**) cream samples stored under different storage conditions. Data are shown as box plots (median, interquartile range, and range). Different letters indicate significant differences between storage conditions within each group (Wilcoxon signed-rank test for paired samples, *p* < 0.05).

FFAs concentrations in cream samples stored under different storage conditions (refrigeration and freezing) are presented as box plots in Figure 2A (raw samples) and Figure 2B (HoP samples).

In raw samples, FFAs concentrations were significantly affected by storage conditions (Friedman test, χ^2^ = 9.0, *p* = 0.011), with a large effect size (Kendall’s W = 0.75). Post hoc pairwise comparisons using Wilcoxon signed-rank tests indicated a tendency towards higher FFAs values after 72 h of refrigeration compared to 24 h of refrigeration and frozen storage, although no adjustment for multiple comparisons was applied. In contrast, no significant differences were observed across storage conditions in pasteurized samples (Friedman test, χ^2^ = 2.33, *p* = 0.31, Kendall’s W = 0.19). Additionally, HoP resulted in significantly lower FFAs concentrations compared to raw samples at all evaluated time points (Wilcoxon signed-rank test, *p* = 0.031 for all comparisons).

Consistent with these changes in FFAs, the distribution of TAGs molecular species was also differentially affected by storage conditions and pasteurization (Table 2). Under refrigerated storage, most TAGs species remained unchanged, indicating a stable lipid profile with negligible degradation in both raw and HoP samples. Only raw samples showed slight but significant increases in short-chain TAGs (SC-TAGs) CN28 and CN30 at 72 h (*p* < 0.05). In contrast, freezing induced significant alterations, particularly in raw samples. A marked decrease was observed in several SC-TAGs (≤34C), together with reductions in CN36, CN44 and CN48, and a significant increase in CN52 (*p* < 0.05). In HoP samples, changes during frozen storage were less pronounced, with only slight increases in medium-chain TAGs (CN40, CN42, CN44, CN46) and CN50, while the rest of the profile remained stable.

### 3.3. Analysis of PV

**Figure 3:**
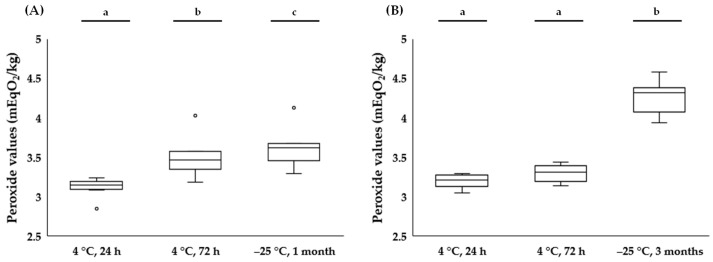
Peroxide values (mEq O_2_/kg) in raw (**A**) and holder pasteurization (**B**) cream samples stored under different storage conditions. Data are shown as box plots (median, interquartile range, and range). Different letters indicate significant differences between storage conditions within each group (Wilcoxon signed-rank test for paired samples, *p* < 0.05).

PV in raw and HoP cream samples under different storage conditions are shown in Figure 3.

In raw cream samples, PV was significantly affected by storage conditions (Friedman test, χ^2^ = 12.0, *p* = 0.002, Kendall’s W = 1.00), indicating a strong effect size. A progressive increase was observed from 24 h to 72 h under refrigeration, followed by a further increase after frozen storage (1 month). Pairwise comparisons using the Wilcoxon signed-rank test confirmed significant differences between all-time points (24 h vs. 72 h, *p* = 0.028; 24 h vs. 1 month, *p* = 0.028; 72 h vs. 1 month, *p* = 0.043) (Figure 3A).

In HoP cream samples, PV also differed significantly across storage conditions (Friedman test, χ^2^ = 10.33, *p* = 0.006, Kendall’s W = 0.86). No differences in PV were found between 24 h and 72 h under refrigeration (*p* = 0.075), whereas PV after frozen storage (3 months) was significantly higher compared to both 24 h (*p* = 0.028) and 72 h (*p* = 0.028) (Figure 3B).

## 4. Discussion

This study presents exploratory insights into the microbiological stability and lipid integrity of the cream fraction derived from human milk under different storage conditions and after HoP. Overall, our findings showed that raw cream contains the same types of microbes that are usually present in raw human milk collected by pumping [11,12]. In contrast, HoP cream samples showed no bacterial growth during storage and exhibited fewer changes in FFAs concentrations and TAGs composition compared to raw cream. However, PV increased over storage time in both raw and pasteurized creams.

Regarding raw cream, no significant changes were observed in the CAN counts across storage conditions. Interestingly, the CNA medium allows the growth of the Gram-positive bacteria that usually characterize the microbiota of human milk under physiological conditions, such as coagulase-negative staphylococci or enterococci [25,26,27]. This suggests that these bacteria remain relatively stable during both refrigeration and freezing of cream, enabling their vertical mother to infant transmission [23].

On the other hand, the MCK medium enables the growth of Enterobacterales and other Gram-negative bacteria that are not members of the autochthonous human milk microbiota but associated with environmental contamination or handling during milk expression, particularly in clinical or hospital settings [11,28,29]. As a consequence, the reduction in bacterial counts on MCK plates observed after frozen storage may be beneficial from the microbiological point of view. Previous studies have reported that total bacterial counts in human milk decrease during prolonged frozen storage (e.g., up to 9 months at −20 °C), with a particularly pronounced decline in Gram-negative populations [30,31]. The higher susceptibility of Gram-negative bacteria to freezing is likely related to the structural characteristics of their outer membranes, which are more prone to damage caused by ice crystal formation and osmotic stress during freeze–thaw cycles than the cell wall of Gram-positive bacteria [32]. Overall, these findings suggest that proper hygienic practices are required during collection, handling, and processing of human milk samples, especially when additional fractionation steps are performed. The presence of high concentrations of Gram-negative bacteria in human milk or human milk-derived products may pose a risk because of the potential harmful effect of the polysaccharides (endotoxin) present in their outer membranes [33]. Anyway, the concentrations of Gram-negative bacteria detected in this study are within the threshold value (<4 log_10_ CFU/mL) allowed by many guides for human milk banking [34,35].

The absence of bacterial growth in HoP cream samples after both refrigerated and frozen storage supports the effectiveness of HoP in ensuring microbiological safety. In the present study, pasteurization was applied to whole human milk prior to cream separation, which is a key factor explaining these findings. HoP is the standard process used in human milk banks and has been widely demonstrated to effectively eliminate vegetative pathogenic microorganisms in donor milk [36]. These results therefore highlight not only the efficacy of this thermal treatment but also the critical importance of strict hygienic handling during post-pasteurization processing steps (e.g., centrifugation and fractionation) to avoid recontamination, as emphasized in previous studies on milk safety systems [37].

This microbiological behavior aligns with the lipid fraction results, in which raw cream samples exhibited a progressive increase in FFAs concentrations during refrigerated storage, particularly after 72 h. This pattern may suggest that viable microorganisms, together with endogenous milk lipases, contribute to ongoing TAGs hydrolysis and subsequent FFAs release. Human milk contains active lipolytic enzymes, primarily bile salt-stimulated lipase (BSSL) and LPL which can retain partial activity under cold storage and promote TAGs hydrolysis [38,39]. In contrast, HoP cream samples showed both lower baseline FFAs levels and greater stability during refrigerated and frozen storage, indicating that heat treatment effectively limits lipolytic activity, largely through the inactivation of BSSL, which is highly sensitive to HoP [40].

Consistent with these lipolytic patterns, the distribution of TAGs molecular species was also differentially affected by storage conditions and pasteurization. Under refrigeration, the TAGs profile remained largely stable in both raw and HoP samples, with only minor increases in short-chain TAGs (CN28 and CN30) observed in raw cream after 72 h. These limited modifications may suggest restricted lipase accessibility to the lipid core, likely due to the preserved structural integrity of MFGM, which acts as a physical barrier to enzymatic hydrolysis [41]. Notably, the relative stability of TAGs distribution despite rising FFAs levels may reflect early-stage lipolysis that has not yet progressed sufficiently to induce detectable shifts in TAGs composition.

In contrast, frozen storage induced more pronounced alterations in TAGs distribution, particularly in raw samples. These changes were characterized by a reduction in several SC-TAGs (≤C34) and specific species (CN36, CN44, and CN48), accompanied by a relative increase in CN52. This pattern is consistent with selective hydrolysis and redistribution of TAGs species, that may be associated with freeze–thaw-induced destabilization of the MFGM. Freezing promotes ice crystal formation and phase separation, which may disrupt the interfacial membrane, increase fat globule coalescence, and enhance TAGs accessibility to residual lipase activity [42]. Similar physicochemical and lipidomic alterations during frozen storage have been previously described in human milk [15]. However, since neither lipase activity nor MFGM integrity were directly assessed in the present study, this mechanistic interpretation should be considered hypothetical. Furthermore, the susceptibility of TAGs species to hydrolysis may depend on fatty acid chain length and positional distribution within the glycerol backbone, with shorter-chain TAGs being more readily hydrolyzed [43].

Conversely, HoP samples exhibited minimal modifications in TAGs composition during frozen storage, with only slight increases primarily in medium-chain TAGs. This enhanced stability may reflect the combined effects of lipase inactivation and reduced microbial load following pasteurization, thereby limiting both primary lipolysis and subsequent lipid rearrangements [40].

Lipid oxidation, measured as PV, showed differences across storage conditions in both raw and HoP cream samples, confirming storage time as a key driver of oxidative changes.

In raw cream, PV increased progressively from 24 to 72 h and further during frozen storage, indicating that refrigeration only partially slows lipid oxidation, while freezing does not fully prevent peroxide formation. Similar trends have been reported in breast milk, where lipid peroxides significantly increase after freezing [44].

A distinct pattern was observed in HoP samples. PV remained stable between 24 and 72 h, suggesting a transient stabilizing effect of pasteurization, likely due to enzyme inactivation [40]. However, this effect was not sustained, as PV increased significantly after prolonged frozen storage. This behavior aligns with findings in infant formula, where thermal processing delays but does not inhibit lipid oxidation, which continues during storage and is modulated by factors such as metal ions, polyunsaturated fatty acids, and vitamins [45,46].

Despite these changes, the overall extent of lipid oxidation remained moderate. In raw cream, PV increased by approximately 14% after 72 h and 19% after 1 month of frozen storage, while HoP samples showed an increase of around 34% after 3 months of frozen storage. Notably, all values remained within a relatively low range (3–4.5 mEq O_2_/kg), suggesting that oxidation did not progress to advanced stages. These values are coincident with the lower-to-intermediate range described for dairy fat systems such as bovine cream and butter (approximately 1–7 mEq O_2_/kg), supporting the interpretation that only primary oxidation occurred under the studied conditions [19,20].

From a mechanistic perspective, PV reflects the formation of lipid hydroperoxides, which are early products of lipid autoxidation. Therefore, the increases observed here are indicative of initial oxidative changes rather than extensive lipid degradation. However, it should be emphasized that some products that arise from secondary oxidation, such as some ketones andaldehydes, which are important indicators of advanced lipid oxidation, were not assessed in this study. Their evaluation, for instance through TBARS or anisidine value, would provide complementary insight into later stages of oxidation associated with sensory deterioration [47].

Although FFAs are generally considered more susceptible to oxidation than esterified fatty acids in TAGs [48,49], no significant correlation between FFAs levels and PV was observed in the present study. Some parallel trends were noted under specific conditions—such as in raw cream at 72 h, where simultaneous increases in FFAs and PV were detected—but this pattern was not consistent across all samples. This suggests that, under the studied conditions, lipolysis was not a primary driver of lipid oxidation and that oxidative changes were likely governed by multiple interacting factors.

Overall, these findings suggest the importance of minimizing storage duration under both refrigeration and freezing conditions to preserve the oxidative quality of human milk cream.

These results reinforce the potential role of human milk-derived lipid fractions as a complementary component within EHMD strategies for very preterm infants. EHMDs, particularly when combined with human milk–derived fortifiers, have been associated with improved clinical outcomes, including reduced necrotizing enterocolitis, lower rates of severe intraventricular hemorrhage, and improved neurodevelopment, as well as reduced healthcare costs in very low birth weight populations [50,51]. While current fortification strategies primarily address protein and micronutrient requirements, lipid provision remains less precisely individualized [52]. In this context, fractionated human milk cream may offer a potential tool to further tailor lipid delivery according to specific clinical needs, such as infants with suboptimal postnatal growth or increased energy requirements, those under fluid restriction requiring higher energy density, or preterm infants with feeding intolerance and physiologically immature lipid digestion and absorption, in whom modulation of lipid load and composition may improve tolerance and nutrient utilization. In addition, this approach may be relevant during the gradual advancement of enteral feeding or when adjusting macronutrient ratios within individualized nutritional strategies.

The present study is limited by several methodological aspects that need to be considered when interpreting the results. The small sample size (*n* = 6 mothers of preterm infants) limits statistical power and reduces the robustness and generalizability of the findings, particularly in the context of human milk-derived matrices, which are characterized by high biological variability. Therefore, the results should be regarded as exploratory and hypothesis-generating rather than confirmatory, especially with regard to TAG molecular distribution and lipid oxidation outcomes Despite the potential clinical relevance of introducing human milk-derived fat fractions into the nutritional management of preterm infants, the exploratory design of the present study does not adequately account for several maternal and lactational factors that may contribute to the observed variability. Although gestational age at delivery ranged from 26 to 36 + 5 weeks and milk samples were collected between 8 and 73 days postpartum, the limited sample size did not allow stratified analyses according to these variables. Since gestational age, stage of lactation, and maternal dietary habits are known to influence human milk lipid composition [53], these factors may have contributed to the interindividual variability observed in the present study. Consequently, future studies should include larger and multicenter cohorts with detailed maternal and clinical characterization in order to obtain more robust conclusions and to better support the potential incorporation of human milk-derived cream into individualized neonatal nutritional strategies. In addition, the use of different frozen-storage durations for raw and HoP cream reflects current clinical practice in human milk banking, where raw milk is generally stored for shorter periods before pasteurization, whereas pasteurized milk may remain frozen for longer periods before use. However, this asymmetric design limits direct comparisons between raw and pasteurized samples at equivalent frozen-storage times and should therefore be considered when interpreting the relative effects of pasteurization on storage stability. Furthermore, no baseline cream sample was available due to methodological constraints related to cream preparation and fat extraction, resulting in the earliest sampling point being 24 h of refrigerated storage. Consequently, very early lipolytic and oxidative changes occurring immediately after processing could not be assessed. This limitation is particularly relevant considering that endogenous lipases in human milk may remain active during refrigerated storage and initiate TAG hydrolysis shortly after milk handling and storage. Recent studies have shown that storage and freeze–thaw conditions may increase FFA concentrations and promote lipid oxidation in human milk during early refrigerated storage [15,54,55]. Therefore, the observed results should be interpreted as reflecting relative changes during subsequent storage rather than absolute changes from the immediate post-processing state. Nevertheless, the FFAs concentrations and oxidative values observed at 24 h remained within ranges previously described for stored human milk under routine handling conditions [15,54]. Furthermore, lipase activity (BSSL and LPL) and MFGM integrity were not directly assessed, limiting mechanistic interpretation of the lipid compositional changes observed during storage. Future research should therefore include mechanistic approaches evaluating enzymatic activity and MFGM stability, together with larger sample sizes to improve statistical robustness and better account for the variability of human milk-derived matrices. In addition, the evaluation of −80 °C storage conditions and the potential use of fortification or antioxidant strategies should be explored to optimize lipid stability and support clinical applications. Moreover, future work should investigate the homogenization stability of this human milk fat-derived ingredient after reincorporation into human milk and evaluate its lipid bioaccessibility and digestibility using in vitro digestion models.

## 5. Conclusions

Holder-pasteurized human milk cream remained microbiologically stable throughout refrigerated and frozen storage and showed reduced lipid degradation compared with raw cream, as reflected by smaller increases in FFAs and a more stable TAGs profile However, PV increased over storage time in both raw and pasteurized samples, although remaining within a moderate range. It should be noted that differences in frozen storage duration between raw (1 month) and HoP (3 months) samples may have influenced the extent of lipid oxidation and lipolysis, and therefore direct comparisons between groups should be interpreted with caution. Overall, our results suggest that human milk-derived cream is as a safe and physiologically relevant lipid source for individualized fortification strategies in preterm infant nutrition while highlighting the importance of minimizing storage duration to preserve lipid quality.

## Figures and Tables

**Table 1:** Bacterial counts in raw and HoP cream samples during refrigerated and frozen storage.

	Storage Conditions	CNA	MCK	Total (CNA + MCK)
		(log_10_ CFU/mL) Mean (SD)	(log_10_ CFU/mL) Mean (SD)	(log_10_ CFU/mL) Mean (SD)
**Raw cream**	4 °C, 24 h	3.53 (1.28)	2.90 (0.88) ^a^	3.85 (1.05)
	4 °C, 72 h	3.29 (1.09)	2.37 (0.43) ^ab^	3.42 (0.97)
	−25 °C, 1 month	3.27 (1.20)	1.59 (0.99) ^b^	3.31 (1.15)
*p* value (Friedman test)		0.401	0.030	0.065
**HoP cream**	4 °C, 24 h	nd	nd	nd
	4 °C, 72 h	nd	nd	nd
	−25 °C, 3 months	nd	nd	nd

Significant differences according to Wilcoxon signed-rank test are indicated by using different letters. can—Columbia Nalidixic Acid agar; HoP—Holder pasteurization; MCK—MacConkey agar; nd—not detected. For contextual interpretation, several human milk banking guidelines recommend total bacterial counts ≤ 105 CFU/mL in raw donor milk prior to pasteurization [24].

**Table 2:** Distribution of triacylglycerols (TAGs) according to their carbon number (CN). TAGs molecular species content expressed as g/100 g fat.

		Raw Creams				HoP Creams		
	Refrigerated		Frozen		Refrigerated		Frozen	
CN	24 h	72 h	1 Month	*p*	24 h	72 h	3 Months	*p*
	Mean (SD)				Mean (SD)			
Short Chain (≤34C)								
CN24	0.036 (0.009)	0.117 (0.109)	0.039 (0.013)	0.846	0.026 (0.015)	0.076 (0.108)	0.032 (0.009)	0.513
CN26	0.086 (0.050) ^a^	0.303 (0.220) ^a^	0.024 (0.014) ^b^	0.009	0.038 (0.019)	0.269 (0.178)	0.028 (0.008)	0.115
CN28	0.082 (0.055) ^a^	0.286 (0.209) ^b^	0.044 (0.036) ^c^	0.002	0.043 (0.023)	0.058 (0.025)	0.032 (0.016)	0.115
CN30	0.111 (0.072) ^a^	0.366 (0.215) ^b^	0.059 (0.065) ^c^	0.002	0.050 (0.032)	0.090 (0.060)	0.047 (0.019)	0.311
CN32	0.264 (0.127) ^a^	0.567 (0.363) ^a^	0.161 (0.084) ^b^	0.006	0.155 (0.091)	0.277 (0.207)	0.113 (0.064)	0.223
CN34	0.682 (0.181)	1.020 (0.431)	0.589 (0.115)	0.311	0.627 (0.081)	0.661 (0.247)	0.599 (0.262)	0.135
Medium Chain (36C–44C)								
CN36	1.213 (0.275) ^a^	1.493 (0.310) ^a^	0.856 (0.267) ^b^	0.016	1.173 (0.149)	1.369 (0.742)	1.050 (0.275)	1.000
CN38	1.171 (0.224)	1.262 (0.417)	1.007 (0.341)	0.311	1.485 (0.350)	1.388 (0.345)	1.382 (0.526)	0.607
CN40	1.848 (0.456)	1.768 (0.959)	1.919 (0.467)	0.513	2.013 (0.468) ^a^	1.846 (0.534) ^a^	2.345 (0.256) ^b^	0.006
CN42	3.314 (0.913)	3.378 (1.156)	3.067 (0.707)	0.311	3.222 (0.586) ^a^	2.879 (0.672) ^a^	3.447 (0.581) ^b^	0.030
CN44	8.348 (1.754) ^a^	8.172 (1.584) ^a^	7.686 (1.589) ^b^	0.042	8.280 (1.325) ^a^	7.669 (1.536) ^a^	8.947 (1.176) ^b^	0.009
Long Chain (46C–54C)								
CN46	12.729 (2.606)	12.772 (2.684)	12.075 (2.286)	0.311	12.592 (2.293) ^a^	12.422 (2.359) ^a^	13.570 (2.220) ^b^	0.042
CN48	16.808 (2.579) ^a^	16.770 (2.204) ^a^	15.735 (2.710) ^b^	0.011	16.678 (2.888)	16.876 (3.595)	17.476 (2.375)	0.223
CN50	20.461 (2.328)	19.772 (1.777)	20.346 (2.156)	0.846	21.382 (1.975) ^a^	21.306 (3.573) ^ab^	20.061 (1.759) ^b^	0.042
CN52	29.747 (7.351) ^a^	28.887 (8.397) ^a^	31.915 (7.601) ^b^	0.006	28.832 (7.981)	29.048 (8.948)	27.161 (6.594)	0.115
CN54	3.657 (3.190)	3.067 (1.604)	4.476 (1.986)	0.184	3.403 (1.035)	3.765 (1.520)	3.711 (1.002)	0.311

Different letters indicate significant differences between storage conditions within each group (Wilcoxon signed-rank test for paired samples, *p* < 0.05). HoP, Holder pasteurization.

## Data Availability

The original contributions presented in the study are included in the article. Further inquiries can be directed to the corresponding author.

## Abbreviations

The following abbreviations are used in this manuscript:

BSSL	Bile salt-stimulated lipase
CFU	Colony-forming units
CN	Carbon number
CNA	Columbia Nalidixic Acid agar
DHM	Donor human milk
EHMD	Exclusive human milk-based diet
FFAs	Free fatty acids
GC-FID	Gas Chromatography with Flame Ionization Detection
HoP	Holder pasteurization
LPL	Lipoprotein lipase
MALDI-TOF MS	Matrix-assisted laser desorption/ionization time-of-flight mass spectrometry
MCK	MacConkey agar
mEq	Milliequivalents
MFGM	Milk fat globule membrane
MOM	Mother’s own milk
PV	Peroxide values
SC-TAGs	Short-chain triacylglicerols
SHM	Skimmed human milk
TAGs	Triacylglicerols
